# On the Form of the Skull in the Natives of the North [of Europe]

**Published:** 1844-10

**Authors:** 


### ART. IX.

1. *Om Formen af Nordboernes Cranier.* Af A. RETZIUS.—*Stockholm* 1843. 8vo. *Mit eenige bekorting vertaald door* J. van der HOEVEN, M.D. Tijdschrift voor Natuurlijke Geschiedeniss en Physiologie, Tiende Deel, pp. 127-80.—*Leiden*, 1843.

*On the Form of the Skull in the Natives of the North* [*of Europe.*] By A. RETZIUS.—*Stockholm*, 1843. *Translated* [*from Swedish into Dutch*] *with some Abbreviation by* J. van der HOEVEN, M.D.; in the Journal for Natural History and Physiology, vol. X.—*Leyden*, 1843.

2. *Ueber die Ureinwohner von Peru.* Von Dr. J. J. von TSCHUDI; in Müller's Archiv für Physiologie; Jahrgang 1844. Heft II, pp. 98-109. —*Berlin*, 1844.

*On the Original Inhabitants of Peru.* By Dr. J. J. von TSCHUDI; in Müller's Archives of Physiology, for the year 1844. Part II.—*Berlin.*

3. *Bijdragen tot de Natuurlijke Geschiedeniss van den Negerstam.* Door J. van der HOEVEN, Med. Dr. Gewoon Hoogleeraar aan de Leidsche Hoogeschool enz. Met vier Platen.—*Leiden*, 1842. 4to, pp. 68.

*Contributions to the Natural History of the Negro-race.* By J. van der HOEVEN, M.D., Ordinary Professor in the University of Leyden, &c. With Four Plates.—*Leyden*, 1842.

WE propose, from the contents of these very interesting essays, to supply for our readers, some contributions to their knowledge of anthropology, and, especially, of that department of the science which relates to the form of the skull in different nations. We take this as the chief subject of our review, not only because of the number of facts relating to it which these essays furnish,* but, also, because the evidence which can be obtained in England, both in books and in museums, is very defective. The general characters of skulls can be discerned, and comparison between them can be fairly made, only by the examination of large numbers of specimens of each form; but our best collections have, at the most, only one or two of each. And this, while it excuses our imperfect knowledge, will enhance, we hope, the value of this contribution to that of our readers.

* Complete accounts will be found of no less than eleven forms of skulls, most of which have hitherto been imperfectly described, namely, those of the Swede, Slavonian, Laplander, Finnlander, three races of ancient Peruvians, and the actual Peruvian, the Negro, and the Kafir.

Before entering on the main subject of his essay, Professor Retzius gives an arrangement of the principal tribes of the human race, according to the form of the skull and face; taking for its basis the comparative length of the skull and prominence of the jaws. This character, indicating as it does the degree of development of the posterior cerebral lobes, and the extent to which they overlap the cerebellum, must be important; for it is a main distinction between man and those of the mammalia, whose cerebral organization is nearest to his, that their posterior cerebral lobes are so little developed, that the cerebellum is nearly or quite uncovered. His first division of the nations of men is into *Dolichocephalæ*, and *Brachycephalæ;* the *long-heads* being, of course, those whose cerebral lobes completely cover the cerebellum, the *short-heads*, those in whom they do not. Each of these main divisions is next divided again, according to the prominence or uprightness of the jaws into the *orthognathæ* and the *prognathæ*. On these grounds, the nations whose skulls he has been able sufficiently to examine, may be thus classed:

Class I. Dolichocephalæ.

Ord. 1. *Orthognathæ*, Gauls, Celts, Britons, Scots, Germans, Scandinavians.

Ord. 2. *Prognathæ*. Greenlanders, various North and South American Indian races, such as the Caribs, Botocudi, &c., Negroes, New Hollanders.

Class II. Brachycephalæ.

Ord. 1. *Orthognathæ*. Slavonians, Finns, and other Tschudisch races, Affghans, Persians, Turks, Lappes, &c.

Ord. 2. *Prognathæ*. Tartars, Kalmuks, Mongols, various North and South American races, such as the Incas, Carruas, &c., Papoes.

If any deduction can be made from this arrangement in its present imperfect state, it is that the most intellectual nations are the Dolichocephalæ Orthognathæ; those, therefore, who, in the high development of the posterior lobes of the cerebrum are most removed from the cerebral character of the brute.

Leaving this, however, let us come to the main subject of the paper. It relates especially to the form of the skull of the Swedes, as compared with those of other European nations, particularly the Slavonians, Finns, and Lappes; and its general result is to prove that the form of the head in Swedes is distinctly different from that in the three neighbouring nations; and that among these three, there are, also, sufficient differences to indicate that they are all of distinct origins.

1. *Of the skulls of Swedes.* Between two and three hundred skulls of Swedes were examined; they were all marked by the same general characteristic form, and the examination of several skulls believed or known to be those of Swedes who lived in the middle ages, proved that this form had been general for at least eight centuries. The general result was that the skulls indicated (by comparison) a remarkable elongation of the posterior lobes of the cerebrum, so that they must not only have covered the cerebellum, but have extended back beyond it.

To describe them more particularly;

"The form of the skull, as seen from above, is ovato-rotund. The greatest length is about one fourth more than the greatest breadth, . . . . very nearly as 9 : 7. On the average the length* from the glabella to the most prominent part of the occipital protuberance is 7·48 inches; the breadth, between the temporal fossæ 4·21 inches; the greatest breadth, which is behind the temples, just in front of the

* All these measurements may be compared with a table hereafter given of the average dimensions of European and Negro skulls.

parietal protuberances, is 5·78 inches; the greatest circumference, above the glabella and the occipital spine, 21·26 inches; the height, from the anterior margin of the foramen magnum to the vertex, 5·31 inches. . . . . The supra-orbital protuberances are strongly marked; and the skull, behind its broadest part, becomes smaller to the occiput, and is lengthened out to the occipital protuberance, which forms a strongly developed roundish process. . . . . The parietal protuberances are absent, or are rounded and but little prominent. The lambdoidal suture forms the posterior boundary of the skull; its edges extend above the surface of the occiput to the lateral surfaces of the skull. The great transverse occipital ridges meet at almost a right angle below and in front of the very prominent occipital protuberance. . . . . When, also, the skull is looked at from the side, the occipital protuberance appears peculiarly large, like a process, which is bounded above by an impression above the angle of the lambdoidal suture, or in the situation where the fontanelle was—a peculiarity which is an essential characteristic of skulls of this form.

"In consequence of this remarkable elongation of the occiput, the external auditory apertures are more forward than in the other skulls; a vertical plane carried through them would always very nearly, and often exactly, bisect the line marking the greatest length of the skull. Another consequence of it is that the temporal ridges do not extend so far backwards as in skulls with a short occiput, but, like the mastoid angles of the parietal bones, lie wholly on the lateral parts of the skull, without reaching the occipital surface." (Retzius, pp. 134-5.)

The same remarkable length of the occiput of the Swedish skull is evident in its base. It has, here, an oval form, and the distance between the apices of the mastoid processes, is only equal to the distance between the anterior margin of the foramen magnum, and the hindmost point of the skull. A line drawn from one to the other external auditory opening would touch that anterior margin. The surface on which the muscles of the neck are attached, and over which the cerebellum rests is nearly level; it lies altogether at the base of the skull. The occipital protuberance which covers in the ends of the posterior lobes of the cerebrum, lies remarkably behind the margin of the surface which receives the cerebellum. The foramen magnum has an average length of 1·41 inch, and breadth of 1·14; and in some skulls is pointed at one or both sides or ends. The mastoid processes are generally large and strong; the pterygoid processes nearly perpendicular.

"The bones of the face, looked at from above, hardly project beyond the cranium; the external orbitar processes are small, and the lower margin of the orbit is almost exactly below the upper one. The prominences of the malar bones lie directly below the external orbitar processes, in consequence of the moderate length of the jaws. The zygoma goes in some skulls straight backwards; in others it forms a nearly regular arch; the greatest distance between the two zygomata is from 5·11 to 5·31 inches. The cheek-bones themselves are flattened externally. The orbits vary in the size as well as in the shape of their external apertures from the oval to the circular form; they are, for the most part, arched obliquely outwards, and the space between their inner margins is usually, as in the other northern nations, wide. The palate is, most commonly, high-arched; and the alveolar process of the upper jaw is deep; it measures from the nasal spine to its lower margin from ·787 to ·983, and its lower margin is a little below the level of the points of the mastoid processes, and level with the middle of the ascending ramus of the lower jaw. The face, therefore, is long; on an average, in male skulls, it measures from the naso-frontal suture to the alveolar margin of the fore-teeth 3·03 inches. The lower jaw, also, is deep and strongly formed; it measures on an average, 2·95 inches from the condyle to the angle, and 1·38 from its lower border to the border of the alveolar process; and thus, as well as by the teeth being, usually, set almost upright, the comparative length of the face is still further increased." (Retzius, pp. 137-40.)

II. *Of the skulls of Slavonians, or Slaves.* The Slavonian skulls that were examined, were those of a Tschech, a Pole, and two Russians; and these were compared with the heads of Professors Presl of Bohemia, and Purkinje, who is a Tschech, and many other living Slavonians. The differences between them and the Swedish skulls were almost constant and uniform. Instead of the long oval form, they presented a remarkable shortness, and an approximation to the quadrangular or spherical form; all indicated a shortening of the posterior lobes of the cerebrum, so that they could scarcely cover the cerebellum.

"Seen from above, the skull exhibits a short or transversely rounded oval form, (*forma breviter ovata*); its greatest length being to its greatest breadth, about as 8 : 7. In three of the skulls the outline resembled a quadrangle with rounded corners, and with the anterior angle smaller than the posterior; in the fourth, (a Russian skull,) it more approached the round form. The bones of the face, like those of the Swedish skulls, when the head was seen from above, appeared but little prominent in front of the cranium.

"The greatest length of the skull is about 6·79 inches; the breadth between the temporal fossæ 4·01, and between the most prominent parts of the parietal protuberances 5·94 inches; the circumference round the glabella, and the most prominent part of the occiput 20·47 inches; the height varies from 5·07 to 6·02.

"The upper surface of the parietal bones is broad and little arched; the occiput is not elongated backwards into a protuberance, growing smaller as it goes further back, but it descends more vertically downwards to the occipital ridges. The parietal protuberances are placed by the beginning of the occiput, (i.e. not the *bone*, but the *back of the head*,) which forms a large low-arched or flat surface, occupying the greater part of the height of the skull, and including the posterior part of the parietal bones, with the end of the sagittal suture; and the whole of the lambdoidal suture. The greater ridges of the occipital bone thus form nearly the lower angle of the posterior boundary of the occiput, or of the base of the skull. The arch of the occiput, just above these ridges, forms an arc, whose height is equal to about half the length of its cord, supposing that cord to be drawn, as in the Swedish skulls, between the external apertures of the ears across the anterior margin of the foramen magnum. The two transverse ridges meet at a very obtuse angle, or pass into each other with a slight bend. Hence the occipital protuberance has the form of a low blunt elevation. The surfaces on which the hemispheres of the cerebellum rest are strongly arched, and the posterior parts ascend and pass into the posterior surface of the occiput. The foramen magnum is like that of the Swedish skulls. The distance between the mastoid processes is from 4·48 to 5·5 inches." (Retzius, pp. 145-6.)

The forehead in profile, in consequence of the frontal protuberances, is nearly vertical, (but in one Russian skull it inclined backwards.) The external auditory passages are behind the middle of the head. The mastoid processes are large, and the temporal ridges reach the surface of the occiput. The form of the face is like that of the Swedish skulls.

III. *Of the skulls of Finns.* The skulls of six Finns, the genuineness of all of which was well proved, were found short, *cuneato ovata* in circumference, with high parietal protuberances placed far back. They differed from those of the Slavonians, in having a smaller and rounder occiput, with straight and flat temples, and an elevation of the parietal bones along the sagittal suture. From those of the Swedes, their differences were as great as those of the Slavonian skulls; and from the skulls of the Lappes, they were distinguished by having a stronger bony fabric, stronger mastoid processes and superciliary prominences, and a longer facial profile; as well as by the round occiput, backward parietal protuberances, and the elevation at the sagittal suture. Their forms were such as indicated a somewhat greater length of the posterior lobes of the cerebrum than the Slavonians possess; though those lobes probably extend only just far enough to cover the cerebellum. Their development in breadth, though greater than in the Swedes, is less than in the Slavonians.

"Seen from above, the skull presents a *forma cuneato-ovata*, with its greatest length about 1-5th more than its greatest breadth. . . . . The average length is 7 inches; the average breadth 5·66; the breadth between the temporal fossæ, 3·93; the average greatest circumference 20·63 inches. From the front it appears flat, in consequence of the position of the margins of the orbits, and the superciliary ridges, but the forehead is vaulted. The outline of the temples is nearly straight, and the temples are flat. The parietal protuberances, which stand out prominently, make an angle at their passage to the occiput, whose arch is greater than in the Slavonians, and forms almost a segment of a circle, . . . and never projects far backwards. . . . .

"Seen from behind, these skulls present a nearly quadrangular occipital surface, which is apparently somewhat higher than broad, (in the Slavonian, the height is only equal to or less than the breadth.) The upper side of the quadrangle lies between the parietal protuberances, the lower between the mastoid processes; its vertical sides are between these points; (in the Swedish skulls the mastoid processes are not within the limits of the occiput, but in front of it.)

"In five (of the six) specimens there is an elevation in the course of the sagittal suture, which is also mentioned in the only account of the skull of the Finns proper hitherto published.* The posterior part of this suture, as well as of the parietal bones, bends downwards at the arch of the occiput already mentioned as forming almost a segment of a circle, and as characteristic of the Finnish skull. The apex of the lambdoidal suture is higher than in the Swede—about as high as in the Slave. . . . . . The great transverse ridges of the occiput are rather lower than in the Slave, but higher than in the Swede. . . . they meet rather above the posterior and lower border of the occiput. The greatest convexity of the occiput is at its middle. . . . . .The part of the occipital bone which covers the posterior cerebral lobes forms about 1-3d of the great round arch of the occiput . . . . The height of the arch, drawn from the margins of the auditory openings round the greatest convexity of the occiput, is equal to about 3-4th of its cord; (the line uniting these openings.) . . . . . . The *conceptaculum cerebelli* is peculiarly developed; and, in five cases (of six) its posterior part ascends. . . . . . The foramen magnum is like that of the skulls already described. The distance between the mastoid processes is, in different skulls, from 4·88 to 5·31 inches: the mastoid grooves for the attachment of the depressors of the lower jaw are deep and narrow.

"In profile the forehead is round-arched. . . . . . . The frontal protuberances stand out and unite in a prominent glabella. The auditory apertures are behind the middle of the antero-posterior diameter. The arch of the occiput is most obvious. The line of the profile of the face is nearly vertical: its height from the root of the nose to the margin of the upper alveolar process is, in five specimens, 2·75 inches; its width between the greatest convexities of the zygomata varying between 5·03 and 5·7 inches. . . . . . . . The zygomata are directed, for the most part backwards; and their lower margins are nearly straight. . . . . . . . The apertures of the orbits are quadrangular, nearly rectangular, and not so high as they are wide. . . . . . . .The palate is but little arched. . . . . . . . The margin of the upper jaw is on a level with the point of the mastoid process. . . . . . . . The lower jaw is not particularly different from that of the Swede." (Retzius, pp. 154-9.)

IV. *Of the skulls of Laplanders.* The characters of these very interesting skulls were examined in no less than sixteen genuine specimens; a number so much larger than any other anatomist has had the opportunity of comparing, (for even Blumenbach had only two in his collection,) that Professor Retzius' account of them must be received with

* By Hueck, in the Bull. Scientifique, publ. par l'Acad. de St. Petersbourg, t. v, p. 316.

deference, though it differs in many respects from those hitherto published. The general result of his examination is to prove that the Lappes, entirely different from the Swedes, belong to the races with short occiputs. Herein they agree with the Slaves and Finns; but they are distinguished from these, chiefly by their skulls being smaller and thinner, with smaller mastoid processes, and, in general, having less marked muscular impressions; moreover, by having the occiput more inclined backwards, and somewhat compressed laterally. They differ also from the Slavonians' skulls, especially in having higher parietal bones, with large protuberances; and from the Finns' in their round temples. The Lappes, therefore, would appear to have the middle lobes of the cerebrum more developed than they are in those other nations, while the posterior lobes hardly cover the cerebellum, and exhibit even a less development in breadth than they have in the Finns'.

"Seen from above, the Laplander's skull presents an outline approaching to the same short oval form as that of the Finns'; while the parietal protuberances are large and far apart, but the lowest part of the occiput is in some degree prominent, and lengthens out the form, and the temporal bones are more arched, and rounder laterally......... The face, as in other Europeans, projects but little before the front of the skull.

"Among the sixteen skulls, the greatest circumference varies from 18·5 to 21 25 inches; in four of them it was 20·66. The greatest length is from 6·1 to 7·08; the average length of the sixteen being rather less than the length of the Finns' skulls...... The greatest breadth is not, as in the Finns' skulls, between the parietal protuberances, but below and somewhat in front of them, in part on the temporal bones, in part on the mastoid angles of the parietal bones. It varies from 5·23 to 6·16 inches, the average being 5·78. The least breadth is from 3·58 to 4·03, the average 3·93 inches. Therefore the longest diameter is nearly 1-8th more than the greatest breadth, and 2-5th more than the least. The greatest average height is 5·07 inches.

"In thirteen of the skulls the lower part of the occiput comes out in a *tuber occipitale*, compressed at the sides. ......The posterior aspect of the skull presents, as in the Slaves and Finns, the form of a quadrangle, with rounded corners, but somewhat elevated at the sagittal suture. The two upper angles are formed by the parietal protuberances, the two lower by the mastoid processes...... The posterior part of the sagittal suture and of the parietal bones is certainly much inclined downwards, but not so much arched as in the Finns, nor so abrupt in its descent as in the Slaves. The apex of the lambdoid suture lies higher than in the Slaves and Finns, and thus much higher than in the Swedes........ The great transverse ridges lie somewhat higher than in the Finns, are only slightly impressed, and meet at a very obtuse angle, without an occipital spine. The surface for the cerebellum ascends in part, and thus passes to the surface of the occiput, as in the Slaves.

"Twelve of the skulls have an elevation along the sagittal suture. But it does not go backwards, as in the Finns; but begins on the middle of the head, extends forwards and, in some skulls, is continued over the upper part of the frontal bone. The temporal ridges reach to the occipital region........ In eleven subjects the grooves for the attachment of the digastric muscles are but little hollowed, but wide and open. The lesser transverse ridges of the occipital bone form small ridges in the neighbourhood of the foramen magnum. In only one subject have the mastoid processes the average size which they possess in the Swedes', Slaves', and Finns' skulls; in all the others they are small: the extreme distance between them is in most 5·11 inches...... The height of the arch, including the occiput, and raised on the line between the auditory openings, is half, or less than half, the length of its cord.

"The horizontal part of the great ala of the sphenoid bone, which receives the middle cerebral lobes, is very broad and flat. The pterygoid processes are directed somewhat forwards...... Seen from the side, the frontal bone in most instances appears arched a little backwards, but in three is nearly vertical. The parietal bones are high-arched, and reach into the occipital region...... The squamous portions of the temporal bones are small and curved. At their union with the great alæ of the sphenoid they especially stand out. The external auditory openings, which in most cases are round, are for the most part behind, but in some at the middle of the length of the skull.

"The orbital prominences are commonly absent, or but little developed. Nearly all the Lapland skulls have their walls with but slight marks of the attachments of the muscles, and are of little strength.

"The profile of the face is but little different from that of the other inhabitants of northern Europe......... As in them, the distance between the orbits is considerable. The apertures of the orbits are nearly quadrangular, and nearly equal in breadth and height; the average breadth being 1·53, the height 1·29 inch. ........The malar bones are small, and their prominences are turned a little outwards. The malar process of the upper jaw is, on the contrary, large, and forms part of the malar prominence........In consequence of the small height of the malar bone, the zygoma covers, in only a few cases, the point of the coronoid process of the lower jaw; in most cases it ends below the zygoma. The greatest arch of the zygoma is formed by the process from the temporal bone; the greatest distance between the two varies from 4·92 to 5·44 inches, and is therefore much less than in the other inhabitants of northern Europe. The alveolar process is low... ....The arch of the palate also is low, and remarkably flat from the front. A line continued backwards from the alveolar margin and in the same direction, passes, in fifteen specimens, to the external auditory openings. ........The lower jaw is in most small and low, its angle very obtuse, its lower margin in many convex. ...... The alveolar margin also is low........The teeth-fangs, in both jaws, are short." (Retzius, pp. 164-71.)

To these, although not entering into the chief part or purpose of the essay, we shall add the description which Retzius gives, from two well-authenticated specimens in his museum :

v. *Of the skulls of Greenlanders.*

"These skulls have a strong bony fabric, strongly-marked muscular impressions, and an ovato-round form, their length being 7·48 inches, their greatest breadth 5·51 inches. In these dimensions they nearly agree with the Swedish; but the breadth in the forehead—which in the Swedes is 4·21—is in them only 3·71 inches. Both the Greenland skulls are knobbed (*knobbelig, knöliga*), and the upper jaw, the malar bone, and the zygoma stand out remarkably from the circumference of the skull.

"The foramen magnum is large: 1·65 inches long, and 1·25 broad. The surface on which the cerebellum rests is large, arched, and remarkably upright; the great transverse ridges of the occiput meet one another at an obtuse angle; the occipital protuberance is round and laterally compressed. The apex of the lambdoid suture lies low, and is very obtuse; the parietal bones arch backwards towards the occipital protuberances; the parietal protuberances are small. The distance between the two external auditory apertures is nearly as great as that between the anterior edge of the foramen magnum and the greatest convexity of the occiput.

"In the West-Greenland cranium there is an elevation along the sagittal suture, which, however, sinks in a little at the middle of the head: in the other cranium the elevation is less, and ceases at the anterior part of the suture. The frontal bone is low, with a slight elevation at the middle, and without protuberances. The temporal ridges reach high up on the parietal bones, and backwards close to the lambdoid suture. The auditory openings are just anterior to the mid-length of the skull, and are small. The mastoid processes are very large: the distance between them 4·92 inches; and the greatest breadth of the skull—5·32 inches—is just above and in front of them. The temporal fossæ are very deep; the temporal portions of the alæ of the sphenoid bone are small, and, as it were, dressed in in front of the part at which the middle cerebral lobes raise up the temporal surfaces. The squamous portions of the temporal bones are large and flat, but prominent at their union with the sphenoid alæ.

"Seen from the front, the frontal bone is small, the external orbitar processes stand very much outwards, the orbital prominences are small, the glabella is elevated: the nasal bones are extremely small, though the breadth between the orbits is the same as in the other northern Europeans. The orbits are large, obliquely placed, with rounded corners, and the external and inferior angle carried downwards: the orbital fissures are large. The height of their anterior aperture is 1·49, the breadth 1·6 inches.

"The upper jaw is high........the malar prominences are large, standing outwards horizontally, cut in an arch below, and descending low over the alveolar portion of the jaw, which is very broad. The distance from the root of the nose to the alveolar margin is 3·14 inches; from the nasal spine of the jaw to the same, ·98. The alveolar margin is broadly rounded, like that which Blumenbach (Decas, v, p. 11,) has described in a Chinese. The palate is low: the pterygoid processes are directed forwards, and small. What is most striking, next to the round prominent upper jaw, is the position of the cheek-bones. Their outer surfaces extend so much from above downwards and *outwards*, that they give these heads, when seen from the front, a pyramidal appearance, such as induced Dr. Prichard to give the name of *pyramidal* to his third class of skulls. The zygomata are very large, and most arched at the middle: the greatest distance between them is 5·68 inches. The ascending rami of the lower jaw are low—2·28 inches; the distance between the angles is 4·52 inches; the chin is round, its depth 1·22." (Retzius, pp. 177-80.)

From these particulars, which we have inserted in unusually long extracts, because the original is within the reach of so few of our readers, we will now point out those general conclusions which are made most probable. In the first place, it is evident, as we have already stated, and as a work by Professor Nilsson, on the History of the Original Inhabitants of the North of Europe, had before shown to be probable, that there is a great difference between the physical characters of the present Swedes and those of the oldest nations of the North, who probably all resembled more closely the Lappes of the present time.

2. The common supposition that the Slavonians, Scandinavians, and Germans are all derived from a common stock is most probably incorrect. The form of the face of the Slavonians differs, indeed, little from that which is general among Europeans; but their skulls, by their approach to the quadrangular or spherical form, differ widely from those of the long oval form which exists in the Swedes and other Europeans, and which Dr. Prichard regards as a general character of the Indo-Atlantic races. And this opinion of the national difference of the Slavonians is confirmed by history, as well as by the manner in which they keep themselves distinct, even when living in the midst of other nations and under foreign governments; a striking example of which is offered by the Tschechi, who have possessed Bohemia for 1000 years; and, notwithstanding their constant intercourse with the Germans, retain still their own language and their national peculiarities.

3. It is further evident that the Lappes and Finns do not belong, as they are usually supposed, to the same race. Their national characters indicate their difference, as well as their respective peculiarities in the forms of their skulls; and it is probable, from the evidence of history and language, that the Finns, Slavonians, and Scandinavians were all nations originally of milder climes, while the Lappes have always inhabited the North. There is evidence* of their having formerly occupied Finnland, a great part of Russia, the south of Sweden, and the whole of Denmark; from which, it would seem, that having been always unwarlike, unsettled, and little civilized, they were gradually driven by more powerful tribes.

4. The anatomical differences of the Finns are also confirmed, as those of the Lappes are, by historical evidence, (adduced from Professor Keyser, of Christiana,) and which renders it very probable that they and their allied tribes, the Esthonians, are direct descendants of the ancient powerful Scythians, who were, to the end of the fifth century, the dominant tribes on the northern side of the Black Sea, but were afterwards dispersed and, for the most part, driven northwards.

5. A comparison of Retzius' description of the Finns' skull with that by Hueck† of the Esthonians' shows great differences between them, but they are of a kind which may be in a great measure due to the differences in the regions they have inhabited, and in their modes of life and social condition. Esthland is a flat country; Finnland, for the most part, mountainous. The Esthlanders were, for many centuries, slaves; while the Finnlanders were, in great part, proprietors of their own land......And they probably lived under different circumstances long before the beginning of our historical records; though their language still agrees so nearly, that the Esthland can only be regarded as a dialect of the Finnish.

6. Blumenbach and most ethnographers have considered the Laplanders and Monguls to be related. A comparison of the skulls of the Lappes with that of a Kalmuk shows that the chief difference between them is, that the upper jaw in the Kalmuk is large and broad, with a large malar process, and deep maxillary grooves; that the malar bone stands far outwards; and that the whole bony fabric is strong.

7. Again, the relation commonly assumed to exist between the Greenlanders and Laplanders cannot be maintained. The description of skulls of the former by Retzius, which agrees with those which Blumenbach and others have given of the skulls of Greenlanders and Esquimaux, proves that they possess a form which is different from any European skull, and constitutes a division in the long series of American races. Retzius' Greenland skulls agree in many respects with the skulls of two Titicacan mummies in his museum.

The mention of this similarity to the skulls of Titicaca will conveniently lead us from Prof. Retzius' essay (on the value of which we need not dwell), to that which stands next on our list, the paper of Dr. von Tschudi, on the Aborigines of Peru. It is but a preliminary essay to a larger work, which he proposes, on the strength of abundant evidence, to publish on the same subject. Its general purpose is to show that among the ancient skulls to be found in Peru there are three distinctly different forms in different localities; and that in the present Peruvians the general form of the skull presents a union of the features of all the three.

VI. *First form of Peruvian skull belonging to the Chincha race.*

"This skull, seen from before, presents the form of a truncated pyramid, whose

* The greater part of which is to be found in Prof. Nilsson's work, entitled Skandinaviska Nordens Ur-invanare.

† De Craniis Esthonum, Dorpat, 1838.

basis is turned upwards. The facial part is small, the orbits transversely oval. The upper jaw descends perpendicularly. The external angular processes of the frontal bone are directed nearly vertically downwards, and are short. The superciliary arches are slightly developed. The arching of the frontal bone from the glabella is slight, nearly vertical to the superciliary arches, and thence gradually extending to the coronal suture. The frontal protuberances are well marked. The parietal elevations are very prominent, so that they form the parts of the skull which project most laterally. At the sides and behind, the parietal bones pass almost perpendicularly to their connexion with the temporal and occipital bones. The posterior wall of the occiput descends perpendicularly to the superior transverse ridge, and then gradually bends obliquely inwards and downwards to the foramen magnum...........The length from the glabella to the point of the occiput opposite to it, which is somewhat above the great transverse ridge, is equal (1 : 1) to the transverse diameter. The inclination of the forehead forms an angle of 68° with the line drawn through the length as above indicated. Camper's angle is 77°. The line from the foramen magnum to the external prominence of the occiput forms an angle of 45°. A plane carried vertically through the meeting of the sagittal and coronal sutures, passes in front of the meatus auditorius and foramen magnum." (p. 99.)

The race to whom the skulls of this form belonged occupied the whole of the coast, which is bounded on the north by Despoblado de Tumbez, on the south by the desert of Atacama, on the west by the Pacific ocean, and on the east by the great coast range of the Andes. Their name is given to them from that of the nation which occupied the coast, between 10° and 14° s. latitude. Their skulls are those which are most commonly seen in Europe, because they are found near the sea-shore of a district many miles in length, covered only by a thin layer of drift-sand. They present, indeed, several varieties of form, such as having one or both sides of the occiput pressed in; but there is no doubt that these forms have been produced by artificial means: the form already described may be easily discerned to be that which is typical of the race by examining a great number of their skulls; and that its peculiar quadrangular form is natural, and not the result of pressure, is proved by its being found in the skulls of children that died unborn; and of whom the skeletons are not unfrequently found in the great burial places of the old Indians of both this and the other races.

VII. *Second form of Peruvian skull, belonging to the Aymara race.*

"Seen from before, this skull is oval: laterally it presents a tolerably regular and somewhat extended arch. The facial portion is large. The orbits are quadrangular; their vertical is equal to their transverse diameter. The upper jaw descends obliquely. The external angular processes of the frontal bone are very strong, short, and directed outwards. The nasal process of the frontal bone is very broad and convex. The frontal bone arches backwards from the glabella, with a tolerably regular inclination, but a greater one than in the preceding form of skull. The superciliary arches are lost; the frontal protuberances are inconsiderable. The parietal bones incline backwards and downwards from their union with the frontal; their protuberances lie deep and are but little developed, so that they are not in the situation of the greatest transverse diameter of the skull, which lies between the upper roots of the zygomatic processes of the temporal bones. The upper part of the occipital bone descends vertically for about an inch from the lambdoid suture, and then bends suddenly and very considerably forwards, and so continues to the foramen magnum with a very slight inclination to the horizon. .......... The antero-posterior diameter of these skulls is to their transverse diameter 1 : 1·3.* The inclination of the frontal bone to the line of the former

* So it is in the original; but, no doubt, it should be as 1·3 : 1.

diameter, (a line carried from the glabella to the point of union of the posterior and middle thirds of the occipital bone), = 45°. The inclination of the lower part of the occipital bone, from the foramen magnum to the superior transverse ridge is only 17°; of this to the upper fifth of the occipital bone is 55°; and that of the upper fifth itself is 85°. The vertical and transverse plane through the junction of the sagittal and coronal sutures passes behind the mastoid processes and through the middle of the foramen magnum. Camper's angle is 68°." (V. Tschudi, p. 99.)

This second race of Peruvians originally inhabited the Peruvio-Bolivian table-land south of the mountain chain of Asangara, elevated 12,000 feet above the sea. From them were derived the dynasty of the Incas, who, in the course of a few centuries, subdued all the other races, and at the same time that they imparted to them their own customs and language, gradually almost obliterated the anatomical differences by which they had been distinguished.

VIII. *Third form of Peruvian skull, belonging to the Huanca race.*

"Seen from the front, the skull presents the form of a quadrangle elongated from below upwards and from before backwards, whose anterior side, extending from one cheek-bone to the other, forms the greatest transverse diameter of the head. The facial portion is large, but shorter than in the second form. The orbits are more elongated, their vertical diameter exceeding by some lines the transverse. The nasal process of the frontal bone is intermediate in breadth between the first and second forms. The external angular process of the frontal bone is thin but rounded, and less arched outwards than it is in the other forms. The frontal bone is small and long; its inclination from the glabella is very great. In many skulls it is concave in the middle, and rises before its union with the parietal bones to a very prominent middle *tuber frontale.* Behind the coronal suture the vault of the skull is somewhat concave. The parietal bones first arch from this part a little upwards, and then descend straight to their union with the occipital bone. The latter bone, between the lambdoid suture and the superior transverse ridge, inclines obliquely inwards; and thence arches very rapidly forwards and inwards to the foramen magnum.

"The antero-posterior diameter, from the glabella to the junction of the sagittal and lambdoidal sutures, is to the transverse diameter as 1 : 1·5.* The inclination of the frontal bone to this line forms an angle of only 23°. The vertical transverse plane through the junction of the sagittal and coronal sutures passes through the part at which the parietal, temporal, and occipital bones are united, and between the foramen magnum and the inferior transverse ridge." (V. Tschudi, p. 100.)

The habitation of this third race was limited to the elevated plains and valleys between the mountain group of Asangara and that of Pasco, which are bounded on the west by the coast-range, and on the east by the inner range of the Andes. They are named after one of the most powerful nations belonging to the race; and the very peculiar form of their skull can be still easily discerned in variously mingled generations.

IX. *Of the skulls of the Peruvian Indians of the present time.* One of the results of the wide-extended conquests of the Incas, descendants of a branch of the ancient Aymaras, was, as already said, such a mingling of the original races, that the succeeding generations nearly lost the typical forms of their skulls. Still, however, all the three forms may be found unaltered in the inhabitants of certain very limited localities. Dr. v. Tschudi states from his own observation, that

"The race of the Chinchas occurs unchanged in some villages on the coast, as well in North Peru as in the valleys of the province of Yanyos; the race of the Aymaras is often found unchanged in the high valleys of Southern Peru; and I

* This also should be reversed, and stand as 1·5 to 1.

have found the most peculiar of all, the race of the Huancas in its unaffected purity in some families in the department of Junin." (p. 107.)

But in the majority of the Peruvian Indians, who have kept from any mingling with Whites and Negroes, the form of the skull, though so different from that of other South American races, that it might easily be taken for a primary form, yet may be traced as developed out of the three forms already described. In the several varieties, also, which this general form presents, it approximates more or less to one or other of the three original forms, and those in whom such approximations are found dwell in parts which were formerly occupied by the original race whom they most resemble.

"The skull (which presents the most general characters of the present Peruvians) approximates in its outline most nearly to the quadrangular form of the Chincha skull. The facial portion is strongly developed; the upper jaw stands rather obliquely; the orbits are quadrangular; the external angular process of the frontal bone is directed considerably backwards. The nasal process of the frontal bone is very convex, and descends perpendicularly; the upper margin of the orbit is swollen and prominent. The arch of the forehead has rather a strong inclination backwards, as in the Aymaras. The frontal protuberances are half lost. The posterior part of the frontal bone and the two parietal bones are formed just as in the Huancas; but at the union of the parietal bones with the upper part of the occipital, the Aymara form appears again very evidently, in that the occiput first inclines slightly from the lambdoidal suture and then arches rapidly and with a greater inclination towards the base of the skull. The antero-posterior diameter of the skull extends, as in the Huancas, from the glabella to the union of the sagittal and lambdoidal sutures; its greatest transverse diameter is, as in the Aymaras, between the upper roots of the zygomatic processes of the temporal bones; they are in the proportion of 1·1 to 1, and therefore, in this respect, the skull most resembles that of the Chinchas."

The evidence of the coexistence of some of the characteristic features of all the original races in the skulls of the modern Peruvians, as well as the existence of the three forms unchanged in some of the natives among whom there is no evidence of any artifices being employed to alter the form of the new-born child, are strong facts in favour of all these forms being natural. Dr. von Tschudi says he has found them all well marked in the fœtal skulls; neither has he, in the numerous mummies of Peruvian children, which he has examined with all their clothes, ever found any trace of an apparatus for compressing the head. He remarks, too, as others have done, that the form of the head, artificial as it looks, is not such as could be given by pressure. The horizontal surface of the occiput of the Aymara race ought to have a corresponding inclination of the sinciput; and the remarkable slope of the Huanca forehead should have had some corresponding trace of pressure on the occiput, if it had served as the surface for counter-pressure. The great length of the Huanca skulls also should indicate that the pressure had been made on their sides; but the inclination of the forhead and occiput are opposed to such a view. On the whole, therefore, the evidence is strongly against any artifice having been employed among the ancient Peruvians to acquire the strangest of the forms which their skulls present; although it is certain that such artifices have been and still are practised in other nations,* and, since the Spanish conquest, have been employed by the Chincha race in Peru.

* We gave some account of the process as employed among the tribes on the Columbia river in the last Number, p. 224.

At the conclusion of this paper, Dr. von Tschudi gives an account of the remarkable occurrence of an *interparietal bone* in the skulls of the children of all three of the Peruvian races. It lies in the situation of the upper angle of the occipital bone, where the parietal bones separate from each other, and is the analogue of the interparietal bone of ruminants and carnivora; it is nearly heart-shaped, its apex lying in the angle of the lambdoidal suture, its base being connected with the occipital bone by a suture which extends between the angles of union of the temporal and occipital bones, a little above the superior transverse ridge. It usually begins to unite with the occipital bone four or five months after birth; the union proceeds from the middle towards the sides, and is not completed at the end of the first year. The existence of this bone was observed in all the children's skulls, (more than a hundred in number,) which were examined, and traces of its suture with the occipital bone are seen in a furrow across this part of all the skulls of these races. The bone might, therefore, if it is found so generally in no other American race, be called the *Os Incæ*, to indicate its remarkable occurrence as a normal formation in one portion of the human race, though it is found only very rarely in any other.

We come, in the last place, to the work of Professor van der Hoeven. He has been long occupied in the study of the natural history of our race, and has published several admirable, both original and translated, papers on it in the 'Tijdschrift,' of which he is joint-editor with Professor de Vriese; and of which we may take this opportunity to say that among the periodicals dedicated to the pure and wide science of physiology, it stands second to none. The present work is the first of a series which he proposes, if it be possible, to publish, collecting in them his scattered anthropological essays, and adding what his continued inquiries have taught him. It has an introduction, containing some good general remarks on the study of the natural history of man, and on the divisions of the human race proposed by former writers on the subject. These we shall pass by; for we wish to keep our first intention of treating only of the forms of skulls; when the learned Professor finishes his essays, and sums them up, we hope to have the pleasure of entering with him into the matter in a more general way; for certainly few are more able than he to give instruction.

x. *Of the skulls of Negroes.* The chief value of the examinations here published lies in the comparative measurements of the skulls of Negroes, Europeans, and Chinese. In the following table the admeasurements given are the averages in the male skulls of ten Negroes and twenty Europeans (namely, five Russians, five Dutchmen, five Spaniards, an Italian, a Scotchman, an Englishman, an Irishman, and a Pole.) The extremes between which the averages lie were in no case wide apart; all the particular measurements are, in the original, given in detail. For the convenience of comparing these dimensions with the corresponding ones of the skulls of the northern Europeans already given from Retzius' paper, we have reduced all the measurements from the decimals of the French metre, in which they are stated, into English inches, reckoning the metre at 39·3708 inches. The *height* of the skull means the distance from the foramen magnum to the vertex; the *length* that from the glabella to the furthest part of the occiput; the *length of the vault* is measured from the naso-frontal suture over the vertex to the margin of the foramen magnum.

| | Europeans. | Negroes. | Chinese. |
|---|---|---|---|
| "Height of the skull . . . | 5·66 | 5·47 | 5·70 |
| Length of the skull . . . . | 7·04 | 6·96 | 7·00 |
| Greatest breadth of the skull . . | 5·47 | 5·11 | 5·43 |
| Breadth of the frontal bone behind the external angular processes | 3·74 | 3·74 | 3·66 |
| Length of the vault over the vertex . | 14·67 | 13·81 | 14·71 |
| Circumference of the skull . . | 20·51 | 19·75 | 20·35 |
| Length of the foramen magnum . . | 1·41 | 1·37 | 1·37 |
| Breadth of ditto . . . | 1·18 | 1·1 | 1·15 |
| Greatest distance between the zygomata . | 5·15 | 5·03 | 5·23 |
| Height of the lower jaw . . . | 1·25 | 1·22 | 1·29 |
| Distance from its condyle to its angle . | 2·58 | 2·40 | 2·67 |
| Length of the lower jaw, from the angle to the symphysis | 3·22 | 3·38 | 3·26" |

(V. d. Hoeven, p. 33.)

From this table it appears that there is no important difference in the average dimensions of the European and Chinese skulls; the greatest differences being in the breadth between the zygomata and in the circumference. But the Negro skull is in nearly every dimension smaller than either; especially, it has less height, less breadth, and less circumference. Its average breadth is less than the minimum of all the European skulls that were examined; and the maximum length of its vault was less than the average of theirs. This conclusion is opposed to that commonly deduced from the examinations of Tiedemann, who, determining the capacities of skulls by weighing the quantity of millet-seed they would contain, believed he could show that the capacity of the Negro skull was not less than that of the European. Measurement is probably a more accurate mode of examination than weighing in cases of this kind; but Van der Hoeven shows from Tiedemann's own numbers, that *on the average* the Negro skulls, which he weighed or had weighed for him, contained 1-20th less than the European skulls; a difference between them which nearly accords with the differences found in the measurement of their several dimensions; and which is amply sufficient to prove that the popular opinion of the inferiority of the negro brain is true.

It appears also that the Negro skull is in the same inferiority of dimensions when measured according to the craniological plan of Carus, in which skulls are divided into three parts, corresponding with the three cranial vertebræ of which they are composed, and distinguished as the forehead, middlehead, and hindhead. Each of these compartments may be estimated in its three dimensions of length, breadth, and height. The breadth of the first is taken at the coronal suture, of the second at the parietal protuberances, of the third at the lowest part of the lambdoid suture. Their heights, in the same order, are measured with callipers from the upper margin of the external auditory opening to the highest part, 1, of the frontal bone; 2, of the vertex; and, 3, to the most prominent part of the occiput. Lastly, their lengths are measured, 1, from the root of the nose to the beginning of the sagittal suture; 2, from the beginning to the end of that suture; and, 3, from its end to the margin of the foramen magnum.

The following table of the comparative average measurements of these dimensions will be useful for more than its immediate purpose :

| | Europeans. | Negroes. |
|---|---|---|
| " Height of the forehead . . . | 4·92 | 4·80 |
| Breadth of ditto . . . . | 4·72 | 4·36 |
| Length of ditto . . . | 4·92 | 4·72 |
| Height of the midhead . . . | 4·95 | 4·92 |
| Breadth of ditto . . . | 5·47 | 5·11 |
| Length of ditto . . . . | 4·95 | 4·75 |
| Height of the hindhead . . . | 4·36 | 4·44 |
| Breadth of ditto . . . . | 4·33 | 4.09 |
| Length of ditto . . . | 4·52 | 4·48 |
| Distance between the outer margin of the orbits | 3·89 | 3·89 |
| Distance between the temporal bones . | 5·47 | 4·8. " |

(V. d. Hoeven, p. 40.)

The rest of Professor van der Hoeven's observations on the Negro skulls are of a general character; chiefly confirmatory of those of previous observers, or complementary to the descriptions already given and well known. Of these, therefore, we need render no account; neither shall we enter upon the subject of his sixth chapter, "the geographical distribution of the Æthiopian race;" for this has been treated at much greater length by Dr. Prichard, upon the evidence of the same authorities as those which are here adduced, and many more besides. We shall conclude with Van der Hoeven's account of the skull of the Kafir (Kaffer, Caffre) tribe, which he describes from five skulls examined by himself, and well authenticated. That which he takes as the type of them, and of which plates are annexed to the work, was the skull of a young Kafir, the son of a chief, twenty-five years old.

XI. *Of the skulls of Kafirs.*

"The height of this skull is about 5·55 inches; its length 7·046; its greatest breadth, which lies between the parietal protuberances, about an inch and a half above the squamous suture, is 4·96 inches. The breadth of the frontal bone behind the external angular processes is 3·46; the length of the vault of the skull 14·76 inches, of which the frontal bone occupies 5·15, the parietal 5·07, and the occipital 4·54 inches. The circumference of the skull is 19·88 inches. The foramen magnum is very oblong, and measures 1·52 by 1·18 inches. The distance between the zygomata is 4·8 inches. The cheek-bones are small. The nasal bones are small, and run to a point; while the ascending processes of the superior maxillary bones are here very broad, and the distance between the orbits is filled up chiefly by them. The nasal bones are not flat, but form an acute angle with each other in the middle line. The superior maxillary bones, below the orbits and beneath the infra-orbital foramen, are deeply hollowed out, and then are continued, small but round, beneath the nose so far forwards that Camper's facial angle is not much more than 70. The teeth. . . .are white, strong, and regular. The lower jaw is long, strong, high in front, (1·19 inches,) not inclining backwards at the chin, but hollowed out below the incisor teeth, and then again projecting at its lower margin. The distance from its condyle to the angle is only 2·16 inches, and the posterior margin of its ascending ramus runs obliquely forwards." (Van der Hoeven, p. 46.)

In its general characters the Kafir skull may be further described as oblong, large, and tolerably high. The nose cannot be flat, as in other Negro races; for in the other skulls examined, although the nasal bones were not so narrow above as in that described above, yet in none were they flat:

"The face is very prominent. The forehead is smooth and round. The occipital bone is in its upper and lateral parts arched and prominent; in the upper middle part flat or even concave, like the parietal bones which at the posterior part by the sides of the sagittal suture are often, and sometimes very remarkably, hollowed out." (Ibid. p. 48.)

It is especially by its greater circumference and greater height that the Kafir skull differs from the common form of Negro skull; and the prominence of the chin is also characteristic; but their general features are the same, and the Negro type is so strongly marked in all the skulls that he examined, that Van der Hoeven does not hesitate to retract the opinion, that the Kafir race must be separated from that of the Negroes, which he had formed on the testimony of travellers.

The acute facial angle, the small size and oblong form, the little distance between the parietal protuberances, and especially between the squamous portions of the temporal bone; and, lastly, the great foramen magnum, all together, make a wide distinction between the Kafir skull and those of the Caucasian races.

In concluding an article on anthropology, in which none but anatomical evidence is adduced in support of the conclusions which are maintained, we are bound to state, on the part of the authors of the essays which we have reviewed, that they have not been wholly negligent of evidence from history, language, and other sources. Both Retzius and von Tschudi's papers are indeed too nearly anatomical; we should hesitate to accept their conclusions, if there be no other evidence for them than that which is contained in these papers; but the former refers to works by Professor Nilsson and others in which he implies that historical evidence in favour of his opinion is contained; and the latter intends his essay only as a preface to a more comprehensive work on the same subject. It would be absurd to neglect or deny the importance of anatomical evidence in questions of this kind; yet, when it stands in opposition to the history of nations, and still more when it is contradicted by the evidence of national language, we think it should give way. We should put a lower value upon it than Professor Retzius does, who, in at least two instances, relies upon it against better evidence from other sources. The Slavonians, whom he regards as a separate race, are proved by language to be most intimately connected with the Goths and Germans on one side, and with the Arian race (including Medes, Persians, and Brahmin-Hindoos), on the other. All the radical words of their speech are found in the languages of these other nations, and their grammatical formation is only a modification of the same principles. Again, as to the diversity of the Lappes and Finns maintained by Retzius, the German writers who have most profoundly studied their history and languages, have come to a nearly unanimous decision, that they are branches of one race. All the evidence for this decision may be found in the third volume of Dr. Prichard's great work. We will also just refer to the notion which Professor Keyser maintains, on the supposed evidence of their history, that the Finns were Scythians. No two races were ever more unlike. The Scythæ were always nomadic, equestrian, warlike, divided into clans, under kings, &c.—the Finns always agricultural, without horses, unwarlike, having no civil government. They had not even a word for *king*, ruler, or potentate of any kind in their speech; and were easily conquered by the Swedes, because they had no political relations between families, and not a notion of clanship.

